# Coping with COVID-19 – Which Resilience Mechanisms Enabled Austrian Nonprofit Organizations to Weather the Pandemic Storm?

**DOI:** 10.1007/s41471-022-00146-8

**Published:** 2022-12-15

**Authors:** Sandra Stötzer, Katharina Kaltenbrunner, Birgit Grüb, Sebastian Martin

**Affiliations:** 1 Institute of Public and Nonprofit Management, Johannes Kepler University Linz, 4040 Linz, Austria; 2 Institute of Business Administration, Strategic Management & Organization, Paris Lodron University Salzburg, 5020 Salzburg, Austria; 3 Institute of Management Accounting, Johannes Kepler University Linz, 4040 Linz, Austria; 4 Department of Health Care, Social and Public Management, University of Applied Sciences Upper Austria, 4020 Linz, Austria

**Keywords:** COVID-19 pandemic, Extreme context research, Nonprofit organizations, Pandemic challenges, Resilience mechanisms, Social and health care

## Abstract

The ongoing COVID-19 pandemic triggered a global crisis affecting the work and partially the existence of businesses, governments, administrations and nonprofit organizations (NPOs). The latter not only faced severe challenges themselves, but also play(ed) a major role in fighting the pandemic, especially those offering services in social and health care. Maintaining service delivery under pandemic conditions to serve the often vital needs of clients requires (organizational) resilience. This concept generally relates to the ability to withstand adversity, to adapt in a turbulent environment and respond to (disruptive) change. Based on a qualitative content analysis of 33 interviews with nonprofit executives, this paper explores the impact of the pandemic on Austrian NPOs active in health and social care in terms of contextual challenges faced. Our study contributes to (nonprofit) resilience research and extreme context research literature as it illustrates how NPOs coped with this disruptive extreme context. Our findings show which resilience mechanisms (i.e. all kinds of resilient behavior, resources and capabilities) were helpful in overcoming pandemic challenges and getting through these hard times.

## Introduction

The COVID-19 pandemic caused an unprecedented health as well as economic and social crisis across the globe. Countermeasures like lockdowns, quarantines, or social distancing to curb the spread of the virus affected the way we live and work, and posed challenges for organizations of all sectors (Brammer et al. 2020; Bailey and Breslin 2021; Plaisance 2022; Sarkar and Clegg 2021). Since the first peak of the pandemic in early 2020, nonprofit organizations (NPOs) have played a major role in responding to this crisis and in mitigating its devastating effects on the local and regional level (Dayson et al. 2021; Kövér 2021; Paarlberg et al. 2020; Shi et al. 2020). NPOs are private formal institutions belonging to the diverse nonprofit/third sector (alongside the business and public sector). Further key features characterize them as non-profit distributing, self-governing and voluntary (Salamon and Anheier 1992). Adding to this structural-operational definition, NPOs are mission-driven *“human-change agents”* (Drucker 1990, p. x) that promote public interests or purposes; their activities are mainly based on values and motivations such as compassion or caring about the community or environment (Anheier 2014). As in many countries, Austrian NPOs – esp. those offering caring and counseling services in social and health care – played an essential role in fighting the pandemic. Particularly in this field, a continuous or sometimes an extended service supply was necessary and partly vital for beneficiaries (Meyer et al. 2021; Millner et al. 2021). For instance, care for the elderly in nursing homes or for people with disabilities in residential facilities had to be maintained despite difficult conditions to ensure their well-being and survival. An expanded range of services and commitment was necessary, e.g., in children’s villages. The young people not only needed the usual care, but also more mental support and help with the initially new experiences of homeschooling. So, while NPOs were essential in managing the crisis – together with government agencies and other public institutions as well as private actors –, they were, at the same time, adversely affected by the pandemic and suffering from major financial and operational impacts (Hutton et al. 2021; Kim and Mason 2020; Kober and Thambar 2021; Meyer et al. 2021; Plaisance 2022). Given their often-crucial role in dealing with the pandemic’s effects (and those of other crises, too), it is a relevant endeavor to explore the mechanisms that enable NPOs to weather such hard times.

The COVID-19 pandemic can be regarded as a disruptive extreme context (Brammer et al. 2020; Rouleau et al. 2021; Sarkar and Clegg 2021), i.e. an environment that is risky, intense and often-dangerous (Maynard et al. 2018) and externally triggered by an extreme event (Hällgren et al. 2018): in our case the coronavirus. Weathering such an extreme context needs a collective response (Mithani 2020). Although disruptive contexts hardly reappear the same way, there can be some principles for coping with disrupted contexts (Christianson et al. 2009; Hällgren et al. 2018). Coping depends on context (which can also generate context-specific challenges and opportunities). A systematic analysis of situational or contextual features (which can be categorized into task, social, physical, and temporal context) can improve understanding of how extreme contexts form or affect organizational phenomena (Bell et al. 2018; Johns 2006), like, i.a., resilience which is impacted by context and depends upon it (Fietz et al. 2021). The ability to respond to threats or extreme contexts, in general, and maintaining service delivery under pandemic conditions, in particular, requires resilience (Bailey and Breslin 2021; Hutton et al. 2021; Mithani 2020). Building resilience has become of high relevance for many organizations as the number and magnitude of incidents increase (Jia et al. 2020). We understand resilience in line with the process- and interaction-oriented general conception of resilience by Williams et al. (2017). Despite the linkages between the different levels of the complex resilience phenomenon, our study focuses on the organizational resilience of NPOs that in short *“refers to the ability to respond productively to significant disruptive change and transform challenges into opportunities”* (Witmer and Mellinger 2016, p. 255).

Research dealing with disruptive contexts from a management perspective is still limited and fragmented (Hällgren et al. 2018). With a focus on the ongoing COVID-19 pandemic, Rouleau et al. (2021) regard this crisis as an opportunity for extreme context research (ECR) and they outline numerous worthwhile research gaps concerning, for instance, the sources of organizational flexibility during a pandemic, and they pose several questions like, e.g., *“What gets normalized (…) how during extended crises?”* (p. 3). Investigating possible answers to this and other questions particularly needs a better comprehension of both resilience mechanisms (like, e.g., slack; Mithani 2020), and of context (which influences resilience; Fietz et al. 2021). With regard to NPOs’ organizational resilience, so far only little research is available (Waerder et al. 2021). Research on NPO resilience mechanisms in extreme contexts is especially needed (Hutton et al. 2021; Searing et al. 2021) as well as studies focusing on institutional differences or similarities across (nonprofit, for-profit and public) sectors (Linnenluecke 2017). For the Austrian context, Meyer et al. (2021) examined challenges for social and health care NPOs during the COVID-19 pandemic and the impact on their relations with government. However, their study only relates to the early phase of the pandemic and does not deal with resilience mechanisms. Thus, to narrow this gap, we investigate the following questions: Which contextual challenges characterized the COVID-19 pandemic as a disruptive context, and which resilience mechanisms helped Austrian social and health care NPOs to cope with these challenges? We base the term resilience mechanisms on the works of Mithani (2020) and of Hillmann and Guenther (2020). Accordingly, resilience mechanisms are instrumental to adaptation and subsume all types of resilient behavior, resources and capabilities that determine a resilient reaction to adversity. For answering our research questions, we opted for an exploratory study design and conducted a qualitative content analysis of the material gained through 33 semi-structured interviews with managers of 14 Austrian NPOs providing services in social and health care.

Our study contributes, on the one hand, to extreme context research^1^ literature: by systematically analyzing the COVID-19 pandemic as a new disruptive context, we enrich knowledge on the different (and so far, only rudimentarily investigated) context (features) of the pandemic. Thereby we deepen the understanding of the specifics of disruptive contexts in general and the current pandemic in particular. This seems beneficial for developing new or improving current coping strategies because *“(…) even if events may never quite recur in exactly the same way, the types of activities that transform chaos into order likely will”* (Hällgren et al. 2018, p. 135). We understand coping strategies as concrete activities (here for managing pandemic challenges) that are connected with resilience mechanisms (which we interpret as the fundamental basis for the activities that can transform or deal with chaos). On the other hand, our analysis contributes to resilience research in terms of resilience mechanisms appropriate for NPOs in disruptive contexts. Appropriateness refers to taking into account the specifics of NPOs. From our point of view, this is necessary and essential because due to their characteristics (esp. with regard to their values and mission as well as their organization and resources) we identified both “universal” and NPO-specific resilience mechanisms. In addition, through an enhanced understanding of the situational features of the current pandemic and a close insight into resilience mechanisms of NPOs, we intend to support (both nonprofit and public) practitioners in better managing future pandemics or other crises.

The remainder of the paper is organized as follows: Next, we briefly cover essentials of (organizational) resilience, provide an overview on the fast-growing literature concerning pandemic challenges and coping strategies by NPOs during the COVID-19 crisis, and introduce our conceptional framework that links ECR and resilience research. In Sect. 3, we outline our research methods. Then, we present our findings in Sect. 4, followed by a discussion and conclusions.

## Theoretical Background and Current Research Insight

### Conceptual Foundations of (Organizational) Resilience

The complex construct of resilience is lacking a consistent definition and is conceptualized differently across several research streams (Duchek 2020; Linnenluecke 2017; Williams et al. 2017), e.g., as a capacity, capability, characteristic, outcome, process, and several others, or a mix of these (Hillman and Guenther 2020 whose systematic review gives a comprehensive overview on the abundance of notions and their attributes). Thus, resilience research is fragmented and also highly context-dependent (cf. Linnenluecke 2017 who reviewed the development of the concept in business and management literature). Basically, resilience is rooted in the Latin term “resilire” which means to jump back in a former position (Guistiniano et al. 2020) and generally reflects the ability to resist adversities while maintaining and adjusting functioning, e.g., in terms of service delivery (Van der Vegt et al. 2015; Searing et al. 2021; Williams et al. 2017). The concept is further characterized by its multi-level nature, i.e., it can refer to individuals, teams, organizations, or broader systems (such as cities, communities or societies) and thus to individual, group, organizational or societal abilities (Williams et al. 2017; Witmer and Mellinger 2016; Raetze et al. 2021). Moreover, resilience can have a static or dynamic nature. Static resilience intends to bounce back to the previous normal – the original equilibrium – and thus contributes to preservation. Dynamic resilience, though, contributes to evolution and assumes that it is not possible to return to the original state; it aims at finding a new equilibrium or even new equilibria (Mithani 2020).

We decided to rely on the broad conception of resilience by Williams et al. (2017) as, on the one hand, its process perspective^2^ accounts for the evolving character of resilience (Witmer and Mellinger 2016), and on the other hand, it highlights that resilience works at the interface between an actor and its environment (Mithani 2020). In their extensive review article Williams et al. (2017) define resilience *“as the process by which an actor (i.e., individual, organization, or community) builds and uses its capability endowments to interact with the environment in a way that positively adjusts and maintains functioning prior to, during, and following adversity”* (p. 742). On the basis of this overarching understanding of resilience, we further direct our focus on the organizational level. Again, notions of organizational resilience are numerous (Duchek 2020; Hillmann and Guenther 2020) and mostly based on attributes like ability to adapt, ability to cope, and ability to reinvent/reconfigure (Hillmann and Guenther 2020). Hillmann and Guenther define organizational resilience as *“the ability of an organization to maintain functions and recover fast from adversity by mobilizing and accessing the resources needed. An organization’s resilient behavior, resilience resources and resilience capabilities enable and determine organizational resilience”* (p. 31). These authors specify resilient behavior in terms of avoidance, conquering denial, acceptance (facing down reality), and embracing paradox. Resilience capabilities comprise anticipation and sensemaking, while resilience resources subsume cognitive, emotional, structural and relational resources. The latter underline that it often takes collective effort or social networks to withstand adverse conditions (Mithani 2020, Kim et al. 2022), which emphasizes the role of social capital as a factor for building organizational resilience (Jia et al. 2020).

We also regard the work of Mithani (2020) as valuable for our study, as he provides a theoretical foundation of resilience modes in extreme contexts. He addresses five resilience modes that contribute to organizational adaption: avoidance, absorption, elasticity, learning, and rejuvenation. Avoidance means evading the threat, e.g., evading a threat of a narrow geographical impact. Absorption refers to the capacity of absorbing the shock or impact of the extreme context while maintaining functionality. Elasticity reflects the flexibility of an organization and its interactions despite enormous turbulences; this mode does not at any time maintain the status quo. Learning corresponds to the modification or the development of new properties, capabilities and mental models like, e.g., new skills, operations, interactions and relationships. In contrast to elasticity, learning involves a time delay. Finally, rejuvenation occurs much later and refers to redevelopment after the original system (or functionality) ceased its existence, which might be the case when an organization has to rebuild itself after experiencing life-threatening events (e.g., due to armed conflicts). Based on his synthesis of organizational research on resilience, Mithani (2020, p. 520) gives an informative overview of numerous mechanisms at both the individual and organizational level that are instrumental to adaptation and relate to each of his five modes. For the mode learning, for instance, he mentions trust and intellectual stimulation at the individual level, and, among several others, the following mechanisms at the organizational level: ability to improvise, access to timely information, capacity to tolerate uncertainty, collaboration, creativity, innovativeness, information sharing, shared vision or slack.

So far, there are only a few empirical studies that deal specifically with the organizational resiliency of NPOs (cf. Searing et al. 2021; Waerder et al. 2021). First, the qualitative inquiry by Searing et al. (2021) reports the experiences of US nonprofits with the financial crisis due to the 2015–2017 Illinois Budget Impasse (which held up payments). The authors’ “nonprofit resilience framework” appears quite universally valid as it maps tactical themes (and subordinate resiliency tactics – here only exemplarily mentioned) in five areas: financial (e.g., cash flow monitor), human resources (e.g., reduce staff), outreach (e.g., improve relations with external stakeholders), programs and services (e.g., reduce service quantity or quality), management and leadership (e.g., leader as example). Second, Waerder et al. (2021) qualitatively studied the contributions of collaborations between NPOs and private-sector firms to organizational resilience of German NPOs in the context of the 2015 refugee crisis. The authors derived the following contributions of such collaborations: concerning resource-based challenges: personnel and financial resources, spatial support; related to conceptual challenges: expertise, dissemination of information, and service expansion; and with regard to emotional challenges: compassion, understanding, and solidarity. Finally, Witmer and Mellinger’s (2016) case study of two healthcare NPOs aimed to identify organizational characteristics indicative of their resilience. They identified six themes that enabled the NPOs to successfully adapt to funding changes or other challenges. These characteristics (or “resilient qualities”) are a strong commitment to the NPO’s mission (i.e., purpose of the NPO as described in its mission statement); improvisation (ability to improvise using existing resources); community reciprocity (mutual and trusting relations with the community); servant and transformational leadership style; fiscal transparency; (a shared perspective of) hope and optimism. In sum, all three studies are not related to today’s pandemic, and all of them emphasize the importance of collaborative relationships for NPOs’ ability to adapt to volatile environments and/or recovering of crises.

### Coping with COVID-19-related Challenges – Research Overview

The COVID-19 pandemic as an extraordinary extreme context has challenged NPOs since its outbreak. Research on pandemic challenges faced by NPOs and on activities or strategies helping them to overcome the obstacles has been fast growing ever since. As expected, most studies we found so far investigate NPOs’ experiences during the first months of the pandemic (mainly spring 2020) and lack a sound theoretical foundation. We identified both empirical and conceptual papers often dealing with specific facets, e.g., pandemic impact on NPOs’ financial stability (Johnson et al. 2021), consequences for nonprofit boards’ roles (McMullin and Raggo 2020) or for human resource management and nonprofit employees (Akingbola 2020; Kuenzi et al. 2021). The latter are relevant to us, as without the commitment of their employees NPOs *“cannot weather the pandemic’s storm”* (Kuenzi et al. 2021, p. 825).

With regard to works that do not explicitly refer to resilience, we identified primarily quantitative research for the US context, like, for instance, the survey by Deitrick et al. (2020) investigating pandemic effects on NPOs in San Diego. They faced disruptions and financial hardships as many were closed (or programs were cancelled) and could not generate income. Accordingly, challenges related to retaining staff, remote working, technology, health and safety, or to staff’s mental health. Helpful for addressing these problems were, among others, reevaluating fundraising strategies, financial scenario planning, technology support, self-care (for leaders and/or their teams) or coaching for leading in ambiguity. Another survey by Kim and Mason (2020) also showed that US human service and arts nonprofits experienced effects on their financing and programs, whereby NPOs with more operating reserves were less affected. This finding underlines the importance of effective financial management and financial reserves to absorb the pandemic impact. One qualitative study by Shi et al. (2020) explored pandemic responses of four homeless-serving NPOs in Texas. They faced problems due to the turbulent circumstances and often-unclear public health guidelines and government mandates. Despite higher demands, containment and mitigation measures disrupted service continuity, and they faced safety concerns, increased financial needs, and logistical obstacles. The authors observed various creative responses by adaptations, innovations (like, e.g., creating “tele” social work processes), and courage whereby all NPOs could maintain mission-critical services.

Another (case) study of a South African NPO by Chikwanda (2020) explored its strategies to maintain effectiveness and survivability during the pandemic. Despite serious difficulties (e.g., cash flow shortages), the author highlights opportunities like the need and inspiration to be more agile, flexible, and innovative. Thus, the NPO could quickly adapt, realign its purpose and set realistic goals. The successful adaptation built on *“commitment to the mission, improvisation, community reciprocity, servant and transformational leadership, hope and optimism, and general transparency”* (p. 685).

We also found several European studies (cf. Kövér 2021) with a focus on the relations of governments with civil society organizations (CSOs). For example, Harris (2021) analyzed the responses of the UK civil society and government and highlighted organizational and governance-related challenges as well as the acceleration of pre-existing trends (esp. online volunteering). For the Austrian context, the mixed-methods study by Meyer et al. (2021) explored how social and health care CSOs and their relations with government were affected during the first months of the pandemic. They identified various challenges due to the first lockdown (enacted in March 2020) and the measures for containing the spread of the virus. Challenging were, i.a., increased customer demands and costs, (partly) income decreases, disrupted organizational practices, dissolved routines, necessary reorganizations of operations and human resources, interaction with public institutions (incl. the uncertainty which costs would be covered), or dealing with psychological stress. Responses by the CSOs comprised, e.g., implementing telework and digital solutions, creating new services or channels to serve clients, applying for financial support from the federal nonprofit fund and for the short-time work program, postponing planned investments as well as using public securities and guarantees for bridging liquidity gaps. Our study differs from the work of Meyer et al. (2021) by its conceptual foundation in resilience and ECR and by its longer investigation period. Their study provides interesting insights into the first months of the crisis and how CSOs were affected by (public countermeasures to fight) the pandemic. Our investigation comprises nearly a year with COVID-19 and thus richer experiences with related challenges (like, e.g., the much stronger wave in autumn/winter 2020 or aspects like testing or vaccination) and coping strategies.

We also identified six articles with explicit reference to NPOs and (distinct forms of) resilience in the COVID-19 pandemic. Table 1 gives an overview on the context and focus of these studies. Some of them illuminate activities and mechanisms that were helpful in overcoming the adversities. Dayson et al. (2021), for instance, found ongoing adjustments and innovations as well as enabling mechanisms incl. tangible factors (like sufficient resources) and less tangible ones (e.g., guiding mission and values) as being valuable for managing the crisis. Another study by Plaisance (2022) reports that the resilience of French cultural NPOs required stable (financial) resources, social/relational capital as well as maintaining human capital (esp. keeping their volunteers). Although human and financial resources were particularly affected by the lockdown, most NPOs could rely on support by their (existing) financial partners and volunteers as relational resources. Finally, Hutton et al. (2021) developed a framework highlighting the interconnectedness of nonprofit and community resilience. They examined NPOs’ adaptive capacities to respond to combined threats posed by the pandemic and hurricanes, and explored factors that may promote or hinder nonprofit resilience (operationalized as being able to continue providing direct services). They reported challenges like strained budgets, increased demand, suspended programs, transition to telework, supply and volunteer shortages as well as fatigue among staff/volunteers. Many NPOs exhibited resilience by using their adaptive capacity to adjust funding streams, reach vulnerable populations, and to change service delivery methods. Thus, they could sustain (or even expand) services and meet community needs, and align strategies and resources to the dynamic context. Overall, NPOs’ ability to withstand disruptions and to recover comprised four aspects: human and financial resources, embedded networks, executive and board leadership, and community participation.

**Table 1:** NPO-related studies dealing with resilience in the COVID-19 pandemic (own compilation)

Study	Context & NPO(s)	Content focus
Dayson et al. (2021)	UK – local community NPOs supporting the elderly in Leeds	Resilience of NPOs/neighborhood networks
Hutton et al. (2021)	USA – NPOs in New Orleans	Nonprofit & community resilience as adaptive capacities for responding to simultaneous threats (by hurricanes & the pandemic)
Kober and Thambar (2021)	Australia – food relief charity	Role of accounting in shaping the NPO’s financial resilience
Maher et al. (2020)	USA – public and nonprofit organizations	Fiscal impacts of the pandemic & responses (with an emphasis on financial capacity and organizational flexibility)
Paarlberg et al. (2020)	USA – community philanthropic organizations (CPOs)	Antecedents of CPOs’ responses to COVID-19 – CPOs activate community resilience
Plaisance (2022)	France – arts and cultural NPOs	Pandemic impact on human, financial & relational resources; NPOs’ resilience requires financial resources, social/relational capital & human capital

In sum, current research gives insights into diverse (and often interrelated and partly context-dependent) challenges caused by the pandemic and corresponding countermeasures for different NPO-types in various countries. Previous studies also list many individual measures NPOs took for handling these challenges. However, only a few studies (but not for the Austrian context) so far deal with (selected) enabling or resilience mechanisms of NPOs that are a prerequisite for effectively weathering the pandemic.

### Conceptual Framework for Linking Extreme Context and Resilience Research

Extreme context research is oriented to understand how organizations cope with (or avoid) extreme events. Such (often unique, unparalleled or unexpected) events like, e.g., natural disasters, terrorist attacks, air accidents, epidemics/pandemics, etc. mostly constitute a matter of life or death and remind of the fragility of life and societies (Hällgren et al. 2018). As they are intense, risky or dangerous (Maynard et al. 2018), extreme events entail significant and high-risk task, social and environmental demands (Driskell et al. 2018). Hannah et al. (2009) describe extreme contexts as environments *“where one or more extreme events are occurring or are likely to occur that may exceed the organization’s capacity to prevent and result in an extensive and intolerable magnitude of physical, psychological, or material consequences to (…) organization members”* (p. 898). So far, ECR represents a nascent and highly fragmented field of research which the comprehensive review by Hällgren et al. (2018) summarizes for the period 1980–2015. These authors developed a typology of three types of extreme contexts: risky, emergency, and disruptive contexts. Examples of risky contexts are oil drilling or firefighting as these are characterized by a near-constant potentiality of catastrophe, while in emergency (and disrupted) contexts that possibility became a reality (as for hospitals or the police responding to actual events). While emergencies allow for preparation (because they relate to organizational core operations), disruptive contexts are triggered by rare extreme events that are independent of core activities and therefore typically catch organizations or communities unprepared, like, e.g., natural disasters (Hällgren et al. 2018), or the COVID-19 pandemic (Brammer et al. 2020; Rouleau et al. 2021; Sarkar and Clegg 2021).

Of course, even in “normal” everyday business, i.e. when organizations such as NPOs are not confronted with an extreme context, they still *“do not operate in a vacuum”* (Vandor et al. 2020, p. 51), but are embedded in a specific context (like their institutional country context, historical context, etc.). Johns (2006) defines context *“as situational opportunities and constraints that affect the occurrence and meaning of organizational behavior as well as functional relationships between variables”* (p. 386). He distinguishes omnibus context (context in general) and – nested within the former – discrete context (particular contextual/situational variables that shape behavior or moderate relations between variables) and proposes dimensions for both. For the discrete context (that is relevant for our study) he refers to three dimensions: task, social and physical context. Bell et al. (2018) also emphasize context in their approach to conduct actionable research with extreme teams,^3^ which always operate in a broader context that influences goals, processes and general functioning of teams. They offer an intentionally broad framework for structuring context in order to systematically identify the characteristics and specific factors that are (likely to be) relevant in certain circumstances. Taking (better) account of context should allow for recommendations that are (more) actionable and serve to show what factors are likely to influence success or failure. The scholars build on Johns’ (2006) dimensions of the discrete context (task, physical and social context) and add temporal context as a fourth category. In short, Bell et al. (2018) describe the four areas as follows: *“The task context originates from the specific work requirements inherent in the completion of performance objectives. The social context includes factors that emerge as a result of having to interact with others. The physical context reflects the arrangement of the physical environment in which the completion of mission-related tasks occurs; significant human-machine interaction issues can also be included here. Finally, the temporal context encompasses features of the team and its environment related to time”* (p. 2748). The authors also specify all four dimensions with numerous examples; for instance, they illustrate the temporal context with examples like extreme time pressure, extended shifts or the duration of the mission, or the physical context with aspects like familiarity, organizational climate or psychosocial closeness/distance to others. We consider the four context areas as a useful concept for our study and for structuring the numerous contextual features of the pandemic. We regard contextual or situational features (for us, these are characteristics of the context or situation in which NPOs found themselves during the pandemic) as relevant as context not only influences the challenges nonprofits had to face, but also their (possible) (re)actions; certain contextual features (as opportunities) promoted their options, while others (as constraints) limited them.

**Fig. 1:**
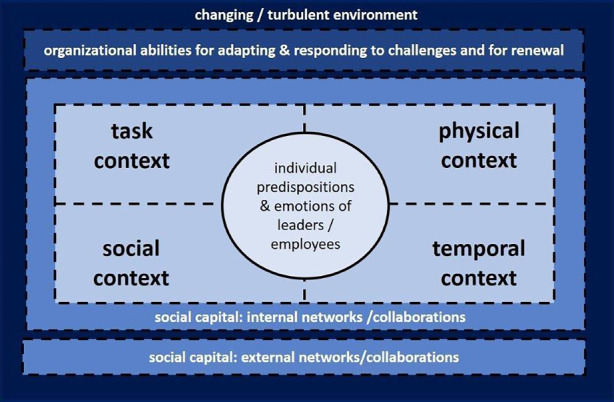
Conceptual framework for linking ECR and resilience research (own elaboration)

As a synthesis of our literature analysis, we developed a conceptual framework (see Fig. 1) that guided our study and links ECR and resilience research. It contains both the outlined context areas of Bell et al. (2018) and considers the changing environment which can entail a disrupted extreme context (represented by the outermost frame) because scholars often relate resilience to the environment (and its characteristics, e.g., uncertain, dynamic or turbulent), and to multiple change phenomena (like disrupted or unexpected events) (Hillmann and Guenther 2020).

In addition, the framework considers the interconnectedness of the levels of resilience. Although our main focus is on the organizational level, the framework deliberately takes into account the intertwined nature of resilience levels (cf. Linnenluecke 2017; Hillmann and Guenther 2020; Raetze et al. 2021). Thus, it includes also the individual level (symbolized by the circle in the center that refers to individual characteristics, behaviors and emotions) and the level of the environment as a superior system in which organizations are embedded and maintaining networks. The (organization-individual) embeddedness in specific contexts takes into account that resilience is also dynamically determined by relations and interactions with various (external) actors (Linnenluecke 2017; Williams et al. 2017). This last aspect refers to social capital^4^, which our framework considers in the form of networks (external and internal networks as we regard both as relevant for large NPOs in social and health care). We decided to integrate social capital because, on the one hand, our review of literature underscored the great importance of networks or collaborative relationships for NPOs’ resilience. The study by Jia et al. (2020)^5^ also emphasizes the role of social capital (in its different forms: structural, relational and cognitive capital) as an external factor for building organizational resilience. On the other hand, considering other types than just economic capital can widen the often-narrow view (on mainly financial resources) to other forms of resource exchanges (based on, e.g., information or reputation).

## Methodology

As our study deals with a relatively underexplored topic, we chose an explorative research design for advancing theory building. The research team conducted one-off semi-structured interviews with nonprofit elites (i.e. top executives, CEOs or board of directors; cf. Ma et al. 2021). Due to our interest in the (multi-level) phenomenon resilience, we were interested in patterns of both individual and firm-/organization-level activities. This qualitative inquiry enabled us to explore the subjective views and practical experiences of nonprofit leaders in order to gain an in-depth understanding of the challenges their organizations experienced during the COVID-19 pandemic and of coping strategies applied by the organizations. We pursued a homogeneous purposeful sampling strategy (cf. Patton 2015; Silverman 2020; Miles et al. 2020) for selecting organizations with similar characteristics based on two criteria that were relevant for us: field of activity (“branch”) and size. First, we consciously focused on NPOs active in social and health care,^6^ which generally meet relevant social problems. Especially during crises, these organizations are a critical asset to their communities (Kim et al. 2022), and the COVID-19 pandemic put them under unprecedented strain making their resilience more important than ever (Barton et al. 2020). As already mentioned, this group of NPOs had a decisive role in managing the pandemic and was at once confronted with severe challenges. Besides, these NPOs provide services in a major field (concerning employment and total expenses) of the Austrian nonprofit sector where collaborative arrangements between NPOs and public institutions – mostly regulated by performance-based contracts – are widespread (Millner et al. 2021; Heitzmann and Simsa 2004; Neumayr et al. 2007; von Schnurbein and Hengevoss 2020). This field of activity includes a wide range of welfare services like support, counseling and caring activities for, e.g., people with disabilities, elderly people, children, individuals suffering from mental illnesses or in need of emergency medical services, and others. Table 2 displays the NPOs included in our sample incl. their concrete service areas. In addition to the authors’ knowledge of the Austrian nonprofit sector, the directories of Austrian umbrella organizations comprising NPOs providing social and health care services were helpful for selecting suitable organizations. Specifically, we drew on data and lists of the “Verband Sozialwirtschaft Österreich”, the “Interessenvertretung Sozialverband” and the “Fundraising Verband Austria” and, in addition, we analyzed information available online about shortlisted NPOs. Of all the organizations contacted, one refused to participate (by simply not answering at all, despite repeated inquiries). Second, size was an important selection criterion for us. We consciously focused on large NPOs in terms of the size criteria (based on income thresholds) of the Austrian law for associations (“Vereinsgesetz 2002”) because the large NPOs in social and health care are not only essential service providers of this relevant industry, but also play an important role in shaping policy and political decision making in this service area (Heitzmann and Simsa 2004; Neumayr et al. 2007; von Schnurbein and Hengevoss 2020). Besides, large NPOs usually differ considerably from small ones (esp. with regard to their degree of professionalization, structures or resources), which is likely to affect their resilience as well. Thus, we decided to focus on large NPOs as a first step.

**Table 2:** Overview of the interview sample (own elaboration)

NPOs	NPOs’ service areas	Interviews
Arge für Obdachlose	Care for homeless people, eviction prevention, employment, etc.	2
Caritas OÖ	Various welfare services for people in need (of care), old people, ill or dying patients, their relatives, people with disabilities or mental health issues, asylum seekers & migrants, families, children, etc.	3
Diakoniewerk OÖ	Various welfare services for people in need of care and accompanying (like people with disabilities or old people), living facilities, family counseling, support for refugees & migrants, health and education, etc.	3
EXIT sozial	Support for people with mental health and social problems (e.g. assisted living, consultation, psychotherapy, mobile support, recreation, etc.)	2
Institut Hartheim	Care and support for people with cognitive and multiple impairments (incl. residential facilities, living and employment opportunities, sports & leisure programs, family counseling for relatives, etc.)	2
Lebenshilfe OÖ	Care for people with disabilities (residential facilities, mobile care, workshops, early support, family support, inclusive employment, etc.)	2
OÖ Kinder-Krebs-Hilfe	Help for children with cancer and their relatives (in financial, organizational, emotional and informative terms)	2
Österreichisches Rotes Kreuz OÖ	Various welfare services for people in need like old or ill people, people who had an accident, children … incl. ambulance & emergency service, first aid courses, blood donation service, 24-hour care, mobile care, day care facilities, assisted living, meals on wheels, palliative care, advice, everyday support, child care & leisure activities, etc.	3
Persönliche Assistenz GmbH	(mobile) care for people with disabilities (support in basic services like personal hygiene or food intake, support with household chores, assistance with communication, mobility & leisure activities, etc.)	2
Proges	Support on health, education & social issues like psychotherapy & psychological counseling, CliniClowns Upper Austria (laughter as therapy), education & training, various prevention offers for children & youth, community health promotion projects, etc.	3
Pro mente OÖ	Support for people with mental health problems or in difficult psychosocial situations (crisis aid, psychosocial counseling, geriatric psychiatry, addiction prevention & counseling & treatment, housing and mobile support, education, training for (re-)integration, etc.)	2
SOS Kinderdorf	Help for children and youth in need (incl. operation of children’s villages, international aid projects, children’s rights advocacy)	2
Volkshilfe OÖ	Various welfare services in the field of health and care for people in need of care (like old people or people with disabilities) & caring relatives, people in poverty and need of support, children, refugees & migrants, incl. residential projects & mobile care, 24-hour care, advice, education, training, work integration, charity shops, etc.	3
Anonymized on request	(no information due to the requested anonymization)	2
*∑ 14 NPOs*	*–*	*∑ 33*

Regarding the selection of interview partners, we assumed that crises are management tasks and thus first contacted the managing directors^7^ (or their offices, based on a website research), and then used a snowball sampling technique by asking these interviewees to establish contact with other suitable (i.e., knowledgeable) interview partners. We attempted to motivate them by assuring anonymity to all participants (and optionally also of the organization, but only one NPO wanted to be anonymized) and offering to provide aggregate results (which everyone was interested in). We always spoke to more than one person per NPO (Table 2 shows the number of informants/one-off interviews per organization). For gaining different perspectives, we aimed at a mixture of managers of different divisions, units or service areas and/or to interview a financial manager. In sum, we spoke to 33 top and middle managers (13 females (39.4%), 20 males (60.6%)) of 14 NPOs (cf. Table 2).

The authors conducted all interviews in German in the period from October 2020 to February 2021 (online via Zoom due to the government’s containment regulations). This enabled us to gain insights into the current and recalled experience of pandemic challenges and coping strategies over nearly a year (from the first lockdown in Austria in March/April 2020 over the reduction of restrictions from May till August 2020 over reinforced restrictions in September/October 2020 till the second lockdown from November 2020 till January 2021). Of course, retrospective reports may provide inaccurate data, e.g. due to perceptual and cognitive limitations, managers’ lack of information, or caused by hindsight bias. However, the ability to recall might be better if informants show high emotional involvement (Huber and Power 1985) which we assume due to the strong impact on almost everyone affected by the pandemic in its first year. Moreover, we conducted the interviews during a peak phase of the pandemic. All 33 interviews were led together by two of the authors (tandem interviewing); this was time-consuming, but proved itself as advantageous both in the case of technical problems and for a structured interview process. The interviews lasted between 20 and 94 min (49 min on average). All interviews were recorded (videotaped with the consent of the interviewees) and transcribed verbatim.

The research team used an interview guideline that was developed in summer 2020 with, altogether, eight open-ended^8^ questions (partly including sub-questions) which the team intensively discussed and pretested (through interviews with two NPO executives which led to small adaptations of single questions and the guideline’s structure). Most guiding questions intended to let the participants speak freely and uninfluenced and were designed by the research team (also because the pandemic was still young and thus publications on it were sparse when we developed the guide). Differently, one question wanted to specify their perceptions of challenges by using visual aids, i.e. we showed them (via screensharing) the four context areas based on Bell et al. (2018). Here the interviewer tandem also used follow-up questions so that the interviewees really referred to each of the four context dimensions. We carried out a qualitative content analysis (Mayring 2010; Krippendorff 2018) of the rich material gained which was separately coded and generalized by two of the authors who afterwards compared and discussed their work. For this paper, we analyzed the answers to the following questions:

- How did you as a leader experience the COVID-19 pandemic?
- To what extent did you perceive challenges in the following areas? (screensharing of picture with task, temporal, physical, and social context – in response to individual inquiries, we gave explanatory examples of the areas)
- How did you overcome the challenges and to what extent did you collaborate with other organizations (NPO, public institutions, companies …)?
- What have you learned as a manager from the COVID-19 pandemic?

In line with nondirective questioning we deliberately avoided naming the topic (i.e., using the term resilience) that might have oriented our interview partners to the nature of the construct we intended to investigate. Avoiding exact words for topics which have a positive connotation (like resilience) might prevent overemphasis or overreporting (cf. Langley and Meziani 2020). In addition, we did not want to influence the answers of the respondents with explanations about our understanding of resilience in the event of (expected) queries. Instead, we communicated the general goal of our study and informed our interviewees that we are interested in their experiencing, coping and learning.

As mentioned before, we interpret resilience mechanisms as the fundamental basis for coping strategies (as concrete activities for managing the pandemic challenges) and base our understanding of resilience mechanisms on the work of Mithani (2020) and also the conception by Hillmann and Guenther (2020). Consequently, we subsume under the term resilience mechanisms all kinds of resilient behaviors, resources and capabilities that advance an organization’s abilities to adapt and to respond to (or cope with) adversities and its ability to renew (reinvent/reconfigure). Thus, for the derivation of resilience mechanisms, we thoroughly analyzed the gained material (esp. with regard to question 3 and 4) and built subcategories in the main category “coping with challenges”. In doing so, we in particular paid attention to those statements by our interviewees where they emphasized what was especially important or helpful for overcoming the respective challenges or the crisis in general.

Looking at the coding process, it was initially guided by two broad main/upper categories: first, the perception of contextual challenges (with the four subcategories task, temporal, physical and social context), and second, coping with challenges (incl. collaboration as social capital). In several passes of first cycle coding (we used a mixture of descriptive coding, In Vivo coding, process and concept coding as well as partly emotion coding, Miles et al. 2020) the two team members individually derived further themes that emerged in the interviews. In terms of pattern coding (second cycle coding; Miles et al. 2020) these were then grouped into (sub-)categories, both for specifying contextual challenges and for coping strategies as well as associated abilities for adapting and responding to challenges.^9^ These results were again discussed and integrated.^10^ The following section now presents our aggregated findings.

**Table 3:** Challenges and resilience mechanisms of NPOs at a glance (own elaboration)

Context area	Main challenges	Resilience mechanisms
Task context	– Expansion & shifting of tasks – Establish crisis management & emergency operation modes (ensure core service delivery & secure liquidity) – Develop workable (operational & change) routines suitable for pandemic conditions – Manifold adjustments (services, funding, procurement, human resource management, communication processes, etc.) – Process/interpret public regulations, implement protective provisions – Lack of planning security due to high uncertainty, dynamics, missing experience – Apply for subsidies/manage short-time work (incl. bureaucracy & emotional injuries) – Maintain/restore a positive morale	– *Shared visions & values – strong commitment to mission (work ethic)* – *Financial strength & organizational slack* – *Supportive relationship networks (social capital)* – Acceptance of reality & ambiguities – Have an eye for the essentials – Flexibility, *improvisation*, creativity, optimism, serenity & pragmatism
Temporal context	– High dynamics (speed of change), time pressure, uncertainty, necessity of ad hoc solutions – Constant accessibility of leaders & work 24/7 – fatigue/exhaustion – Conflicting conditions: – Overtime vs. no or short-time work – Short information & implementation cycles vs. (too) long waiting times – Frequent, often unclear regulations & guidelines from several public authorities: – Constant adjustments & multiple work – (Often bad) timing & way of information (via media)	– *Strong commitment to mission* – *High motivation & work ethos* – Physical & emotional fitness (incl. breaks & personal sources of energy) – Acceptance of reality & ambiguities – Have an eye for the essentials – Flexibility, *improvisation*, serenity & pragmatism
Physical context	– Development of hygienic protective measures and spatial provisions – Development or expansion of digital formats – Setting up effective home office (incl. IT & gender issues)	– *Financial strength & organizational slack* – Flexibility, openness to change & new technologies – Creativity & innovativeness – *Improvisation/problem-solving skills* – *High level of intrinsic motivation & work ethos (ethic of care)*
Social context	– Maintaining confidence & mental wellbeing – Enabling effective leadership at a distance – Developing/maintaining a sense of community – Specific leadership challenges: – Gender issues – Risk groups in the workforce – Onboarding of new staff	– *Supportive relationship networks* – *Organizational slack* – *Shared visions & values* – *High levels of intrinsic motivation & ethic of care* – Dynamic redefinition/reinterpretation of organizational identity – Individual properties (e.g., optimism, physical and emotional fitness)

## Findings

In the next subsections, we present the identified pandemic challenges and assign them to task, temporal, physical, and social context. As mentioned before, these context areas are often interrelated; as a result, the outlined challenges in part overlap or can be assigned to more than one area. Nonetheless, the context areas serve as a common thread for a structured presentation of our manifold results concerning the interviewees’ experiencing of the pandemic, reported coping strategies and lessons learned. In sum, our findings provide an overview of the main (contextual) challenges, and in addition, we finally summarize which resilience mechanisms helped the NPOs managing the pandemic as an extreme event.

### Task Context

The COVID-19 pandemic severely affected NPOs’ task context and entailed a lot of extra work. Especially at its beginning, tasks *“exploded”*^11^ (IP 26)^12^ as the pandemic made new and urgent tasks necessary like, e.g., setting up crisis management or applying for grants. This increase also led to a crowding out of other duties because *“COVID devours daily operations to a large extent.”* (IP 9) Suddenly, nonprofit leaders’ job content was *“all about COVID-19”* (IP 22), and assessing the overall situation was difficult, not least because of the often missing and sometimes contradictory information. Due to this expansion and shifting of tasks and because of ambiguities, a lack of planning security as well as missing experience with pandemics, the interviewed executives consistently perceived the situation as very intense and demanding. One told us: *“The work has multiplied. I really have to say that the last year was one of the most intensive (…) I could have never imagined that you can work so much.”* (IP 32)

Another person summed up the uniqueness of the current pandemic as follows: *“This crisis differs fundamentally from all previous crises in that it affects the entire organization, including all internal procedures and processes in a new, previously unknown way.”* (IP 6) Consequently, it was a major task to develop workable operational routines suitable for pandemic conditions. This involved a lot of reorganization and adapting service delivery as well as administration and management. In relation to the provision of services, this meant establishing an emergency operation mode: *“The big challenge was to switch our different services to an emergency mode from one day to the next (…) such an abrupt change, from 100 to 0 (…) and to accompany the people well in this process.”* (IP 4) Simultaneously, it was then challenging to reboot operations. The alternation of lockdowns and phases of eased restrictions (from wave to wave) made it necessary to switch between the modes of shut down and reboot. It became important to develop change routines and – after the initial “shock” and as the pandemic progressed – to cope with the parallel operation of “regular” service provision and new (online) services. In addition, NPOs had to determine and provide core services and they developed – wherever possible – online services as a substitute for face-to-face services for their clients (of course, this new development also involved challenges in the physical and social context). However, the possibilities to offer online services were often limited, since the services in many areas of social and health care represent *“relationship work”* (IP 30), i.e. personal services that live from (direct) personal contact.

Administrative and management issues primarily involved a lot of information processing, interpreting new regulations and implementing the required protective provisions (especially hygienic and protective measures for both clients and employees in order to safeguard their well-being). These necessitated adjustments concerning procurement (mainly of protective material and IT equipment). Another crucial issue was securing liquidity and financing (above all finding alternative funding sources to compensate for revenue declines, negotiating with public financiers, gathering information in order to (try to) understand the complicated public funding system, or applying for the new “NPO fund”^13^). One challenge was raised particularly frequently and emotionally: short-time work.^14^ The main difficulties related to finding and processing information about this new and intricate system, since at first no one knew how the subsidy actually works. Once the NPOs had finally built up the necessary know-how (through a lot of extra work and personal commitment as well as the exchange of knowledge, problems and experiences in networks), they suffered from the associated high administrative effort involved in submitting applications and accounting for short-time work. In addition, some NPOs (especially in elderly care) did not even want to send their (much needed) employees on short-time work, but were forced to do so by public financiers. Some classified this decision as *“cold financial compensation on the backs of organizations and employees”* (IP 28) which also triggered issues within teams. Two selected quotes shall illustrate problems in this regard:

*“The worst challenge for the entire organization was managing short-time work (…) and the innumerable ambiguities associated with it (…) It was an unparalleled chaos and placed extreme demands on our organization and employees. All the bureaucracy actually dragged on until fall.”* (IP 14)

*“There was an instruction from the province [of Upper Austria] that we had to send employees on short-time work. My response was: we don’t need that, I need all the people, there’s a lot to do. Then the message came that we still have to (…) so we looked for who could go on short-time work, without which employees we could most likely do. (…) this led to great problems because it contained a very high potential for personal slights. Then there were the ‘more important’ and ‘less important’ employees and that was difficult to deal with in terms of group dynamics. This caused injuries that have still not been fully resolved.”* (IP 12)

Pressing tasks in human resource management were the following: reorganizing teams, addressing manpower needs incl. staff shortages (due to illness, quarantine, care duties or forced short-time work), and often at the same time^15^ applying for short-time work and implementing a corresponding payroll accounting, establishing shift work (to reduce contacts and ensure service delivery), as well as developing recruiting, onboarding and training processes in virtual settings (most respondents felt that such online substitutes reduced quality). In terms of leadership, it was also essential to maintain (or restore) a positive morale among employees and volunteers who often suffered from the extra workload and the high uncertainty. Overall, modified information and communication processes and increased coordination were necessary, as well as ad hoc problem solving and (re-)organization. One executive summarized his role like that: *“My job is: I am a troubleshooter.”* (IP 8)

Many interviewees also emphasized challenges (for themselves, but also for their employees and volunteers) in terms of information supply and communication of the applicable regulations (see also subsection 4.2 on timing issues). Two final quotes shed light on this problem (which relates to both the task and the temporal context) in connection with frequently changing tasks and communication content:

*“Every time we felt we had finished work on one of the public regulations, then the next one came along and we had to throw away half our work. (…) And we had to say: dear colleagues, yes, that’s what we wrote the day before yesterday; great that you looked at it and implemented it. But we’re going to do it differently now.”* (IP 9)

*“The difficult thing was that we communicated things that were two days later no longer valid. And accordingly, you had to constantly update all employees. This initially caused some irritation and disruption (…) but fortunately, there was also a lot of understanding from the colleagues, because it was new territory for everyone.”* (IP 3)

### Temporal Context

Without exception all interviewees stated that the temporal context was, especially in the early stages of the pandemic, extremely challenging and intense due to extended shifts and overtime hours (Monday to Sunday), high time pressure as well as constant accessibility causing eroding boundaries between family life and work. On behalf of many others, one person said: “*There is no time frame; my working hours were unlimited.*” (IP 17) In addition to the general uncertainty, dynamics, and low predictability, governments and public authorities contributed to the necessity of ad hoc problem solving under pressure as their frequent (and often unclear) regulations were mostly communicated at short notice. While many NPO-executives criticized the chaotic actions of public authorities, some interviewees understood the also challenging situation of public actors which were “*crazy busy with themselves*.” (IP 26) Several managers described how the short-term nature of information (often via the media) and orders increased the relevance of quick reactions and flexibility. The following statements illustrate that challenge:

*“At the peak of the crisis, we received new instructions from both the regional and district administrative authorities nearly every hour, and in addition, new information from the federal government came every day, plus all the media coverage … It was sometimes not possible to react adequately to everything or to implement every policy (…) because of the speed.” *(IP 12)

*“Unfortunately, it was (…) almost always the case that decisions were made at the weekend, and that we found out about it from the media before we got an official information. That means that we went to work on Monday and the phones, the lines were running hot because the relatives had questions, of course. And we have had little time to prepare. (…) Then you still have to wait until the regulation is there, i.e., we lose a few more days. And yes, that makes it very challenging to react really quickly.” *(IP 22)

As mentioned in the last quote, and in contrast to the tight information and implementation cycles, for many NPOs waiting times were demanding (concerning administrative decisions and official letters, but also test results or long delivery times for important equipment). These conflicting conditions (i.e., high speed and pressure versus grueling waiting times) led to tensions and made it difficult to (re)structure and coordinate work processes or find new work rhythms (especially when working in home-office with initially a lack of structure). In addition, leaders had to take into account that their staff was differently challenged: on the one side, they had to send employees on short-time work, while on the other side, there also were *“employees who accumulated vast amounts of overtime. (…) For lack of alternatives, we have committed massive violations of working hours in a number of facilities during the crisis. (…) knowing that they won’t be able to stand that for long and without a real perspective as to when things will get better again. (…) Some were burdened in a way that I as a leader would normally never be able to take responsibility for.”* (IP 14) Consequently, the longer the crisis lasted, the more signs of fatigue became apparent and common (both for managers and employees), also because there was no end in sight and the future so uncertain. However, it should also be mentioned that some top executives told us that their schedules were more relaxed since the beginning of the pandemic. For them the lockdowns also had positive side effects in terms of slowing down: since many business trips and events were cancelled, their representation and networking duties were also reduced.

Several leaders wondered how long they and their staff could bear the burdens caused by the pandemic. They agreed that their extraordinary effort is only possible for a limited time. It was all the more important to have time outs and use the summer break for both relaxation and intermediate reflection. In light of the high dynamics and speed of changing conditions, constant adaptation, flexibility and decisiveness were necessary. Several interviewees highlighted that reacting quickly and communicating decisions clearly were key for conveying security to stakeholders and lifting the spirits. It was also considered essential to structure the time together well. Acceptance was also helpful, not least accepting that there is hardly any planning certainty or predictability, because *“if you knew everything, it wouldn’t be a crisis.”* (IP 2) Faced with uncertainty and endless work, executives need the courage to leave gaps and dare to make decisions based on not only imperfect, but ambiguous and volatile information. With regard to the short-term, changeable and sometimes impractical demands from public authorities, pragmatism was important and *“keeping a level head.”* (IP 12)

### Physical Context

Two aspects were particularly challenging concerning the physical context: first, the (largely legally prescribed) development of hygienic protective measures and spatial provisions, and second, the establishment of home office and digital (service and working) formats. The hygienic measures comprised protective materials (such as facemasks) and distance regulations, which in some cases also made spatial changes necessary (like structural adaptations such as plexiglass protective walls, or separation of common areas). Regardless of the (considerable) cost factor, it was often not easy to implement the respective regulations because of their technical and practical feasibility or due to material shortages:

*“Especially in the initial phase, it was a major challenge to reconcile the legal requirements with the missing resources. (…) there were times where it was already clear that we must, we have to work with mouth and nose protection (…) and they simply did not exist. (…) We then let our employees in our facilities work with self-sewn, unsuitable masks, because there was simply nothing else. (…) Of course, the physical work environment was not and is not geared towards this crisis. (…) In the last few days, we have been particularly concerned with the delivery of unsuitable masks by the state and the federal government. Here, too, we had employees with unsuitable masks in action during a real pandemic.”* (IP 14)

In addition, the changed physical context (with new and comprehensive hygienic protective measures) was not always compatible with the prevailing professional ethos of NPO employees and volunteers for whom it was important to still guarantee empathic and caring attitudes towards clients. This dichotomy of (protective) distance versus (mostly intrinsically motivated) care ethics which is often based on human closeness and direct contact led to tensions and conflicts (and thus is also related to social context).

The new development or expansion of digital formats became suddenly a necessity for two reasons: on the one hand, many NPOs were not able or allowed to continue their (analog) service provision. Consequently, some leaders, employees and volunteers got very creative and designed as well as implemented new online services (e.g., online therapy sessions via computers or smartphones for clients or virtual visits of relatives for residents through tablets) within a very short time. These innovations entailed opportunities (also for the future after the pandemic), but the limits of online service provision became apparent, too (especially regarding normally direct personal services). On the other hand, the prescribed protective measures and legal regulations made it necessary to switch most of the internal collaboration and exchange to online formats. Especially video conferencing (e.g., via Zoom or MS Teams) and instant messaging (e.g., internal WhatsApp groups) were booming and most interviewees commented on associated advantages, but also disadvantages like problems due to the fact that informal communication fell sharply and that the sudden distance to colleagues impacted the team spirit. Again, the link between physical and social context is apparent. In this respect it is a challenge to create substitutes and new modes for developing identification or for onboarding and integrating new employees. It was a challenge how to *“promote gossip online (…) given the physically different work environment (…) Around ten years ago, we introduced an employee breakfast (…) and we just transferred that to Zoom (…) so that we can preserve it in some form, because this facilitates a relatively large flow of information (…) and is important for cooperation.”* (IP 23)

With regard to internal working routines, setting up effective home office was a major issue for most leaders and the affected (office) workers.^16^ But there also were exceptions, namely in those NPOs whose leaders proactively shaped digital change already before the pandemic outbreak (by, e.g., a recent IT modernization or a changeover to digital files). For the others (those without so much own initiative in advance), IT procurement problems were massive during the first months of the pandemic as demand for, e.g., webcams and headsets, increased rapidly worldwide, causing bottlenecks and long delivery times. Most of these procurement issues could be solved over the months, but, of course, the provision and maintenance of modern IT infrastructure required considerable (financial and personnel) resources.

Naturally, not all employees and volunteers were equally skilled and open to new technologies, causing sometimes resistance and necessitating a lot of training, technical support, and remote maintenance. In addition, (physical and other) working conditions at home differed significantly. For example, the performance of the private internet connection is important, but also the living and family conditions affect the effectivity and efficiency of working remotely (e.g., apartment as well as family size influence whether someone has his/her own study room at home, or at least a quiet and undisturbed corner for a small workplace). Here gender differences became obvious as not only many nonprofit leaders are female, but also disproportionately many employees. Women in particular were multiply burdened during the pandemic due to closures of schools and other childcare facilities or day care centers for older relatives. As a consequence, many women suddenly working at home (with often less or worse equipment than in the office) had not only no (or little) rest to work undisturbed, but also additional care obligations for relatives and especially children who frequently needed help with homeschooling. The following quotations intend to exemplify this problem from the point of view of two female executives:

*“I have two children, one is ten, and one will be four in two weeks. I hate home office. I miss the office. (…) When someone says: ‘Home office is great’, then my first questions is: ‘Are there children?’ Everyone I know, who has children, doesn’t think that working from home is that great. So, I make sure that I am in the office as much as possible.”* (IP 29)

*“We are a strongly women-dominated team, many of us have children (…) and we are fed up with home office. I think every working mother knows that working from home can be fun, but when the kids are home schooling at the same time, it’s hell. (…) I now strongly notice this uncertainty in our team, that everyone says, that she doesn’t want anymore, she can’t anymore. And the notion of again sitting at home with the children and doing the work on the side is something that in particular our female colleagues find very stressful.”* (IP 2)

### Social Context (Including Supportive Relationship Networks)

In general, and especially in this context area, the pandemic acted as a “burning glass” and indicator of individual differences, strengths and weaknesses of employees, leaders and leadership approaches. Everyone (employees, volunteers, clients, relatives of clients, executives, etc.) was affected (albeit to varying degrees) and reacted differently. Emotions and mental issues were an all-pervasive factor, especially during the first months of the pandemic (in spring 2020) when there was a lot of uncertainty (e.g., regarding the danger of the disease) and fear to face. It was a great challenge for all managers to deal well with (their own and others’) emotions and mental issues in order to maintain confidence and mental wellbeing. In this regard, interviewees told us:

*“(…) at the employee level. They reacted split (…) between the positions of that is all not so bad, that is grossly exaggerated, to the point of mortal fear. I had employees in fear of death who were no longer able to work at all; in middle and upper management, too. That was a big problem, because when these people are absent, I can’t say, ‘stay at home and get you to safety’, because business has to go on.”* (IP 12)

*“It was part of my responsibility as a leader to act a bit soothing and tell them: ‘Guys, don’t get hysterical! We can do it’.”* (IP 5)

*“I think it makes a lot of difference not to panic (…) because we carry it on, to the employees (…) and also to the clients where we are again required to remain stable (…) stable and realistic (…) but also to remain authentic and honest. (…) just to say (…) ‘we don’t know how long the lockdown will last or when normality will be back.’ But to make it clear that we are there anyway.”* (IP 11)

Nevertheless, the later phases of the pandemic (i.e., autumn and winter 2020/21) with new waves and repeated lockdowns were also demanding because, in view of the long duration of the crisis, many employees and volunteers showed signs of exhaustion. Besides, the long-lasting psychosocial distance was perceived as challenging. This was due, on the one hand, to the pandemic containment and mitigation measures (incl. isolation in home office and social distancing rules), and on the other hand because of the fact that there was hardly any time for maintaining contacts or social affairs. The importance of personal (face-to-face) contacts and analog relations became more than evident – important for mental hygiene as well as for team cohesion and working climate (and, of course, for the wellbeing of clients and service quality). Many executives and employees missed interpersonal contacts and “yearned” to return to their offices. We often heard it that digital formats cannot (or only restrictively) substitute face-to-face contacts, as the following quotes exemplarily demonstrate:

*“Experiencing face-to-face contacts and intuitions are vital. (…) In my opinion, a purely digital support is not possible. You just need the analog, you need to meet people, you need to get a feeling, you need the atmosphere that arises in a meeting.”* (IP 5)

*“We are social beings. (…) I can also say it in everyday language: we thirst for analog personal meetings. (…) And one also saw very clearly (…) that the future will not take place in the digital world alone (…) that is definitely a realization, that we need both.”* (IP 28)

Developing a new leadership style was a main challenge for executives under pandemic conditions, i.e., the question how to enable effective leadership at a distance in times of stay-at-home orders, home office, social distancing, and (predominantly) digital communication replacing face-to-face contacts. Linked to this challenge is also the question how to develop and maintain a sense of community in the workforce or in specific teams (e.g., care team in a specific residential facility). Again, it was crucial to find ways of coping well with emotions and mental issues, to be a self-confident leader, and to have, develop and convey faith or optimism, in order to support employees in this challenging time. Moreover, many interviewees stressed the importance of regularly thanking and acknowledging the employees and volunteers, and to deal well with their different individual characteristics and conditions. Finally yet importantly, it was also a relief for some executives to have (even more) trust in the competencies and problem-solving capabilities of their employees. Selected quotes illustrate some of these aspects:

*“We are actually a very relaxed company. The executives, especially the department heads, can actually act very independently. COVID has changed that, it no longer works. So, everyone has to follow the same rules. In other words, this looseness or ease in leadership is gone. It’s really, um, I don’t mean to say dictatorial now, but maybe that’s the right word to use to imagine how it has to go right now.”* (IP 29)

*“You have to switch your (leadership) mode; you must not believe that you can continue leading as usual. It won’t work. A different form of leadership is needed; in particular I have to assist my colleagues more immediately.”* (IP 5)

*“(…) that was my job: a lot of internal control, a lot of human resource management, a lot of ensuring that the mood doesn’t tilt, that you give people security and structure. (…) orientation, clarity, and structure are extremely important. Always! Indispensable in normal times and even more in times of crisis. If you are not clear and people cannot orientate themselves on you, then the ship begins to shake. (…) And that also turned out to be very good: not too much information, but rather little in a targeted manner and then clear and precise.”* (IP 8)

Overall, we identified (at least) three specific leadership challenges: the first relates to gender issues (due to the predominantly female workforce). As already outlined in Sect. 4.3 (physical context), primarily women had to reconcile work and family life and were heavily burdened or even overloaded. Consequently, both female employees and leaders often represented particularly stressed groups of the NPOs’ workforce. The second issue refers to high-risk staff, i.e. employees who belong to (health) risk groups (e.g., diabetics, overweight people, or employees/volunteers with other pre-existing illnesses) and especially volunteers being older than 65 (as older age implies a higher risk of a serious course of the disease). Thus, these groups or team members had to be protected and were often no longer able or allowed to work (and that complicated the personnel situation and roster creation). The third specific challenge relates to onboarding of new employees or volunteers, their training and integration into the team. Although online substitute formats were designed, the interviewees perceived problems in this area: *“And it was very, very difficult (…) to hire someone new. (…) such an onboarding (…) it works via video, yes, because it has to work, but that’s not entirely smart, I think. You don’t notice anything, and then you are in the company for two months and have not seen anyone in person.”* (IP 17)

#### Supportive Relationship Networks.

**Fig. 2:**
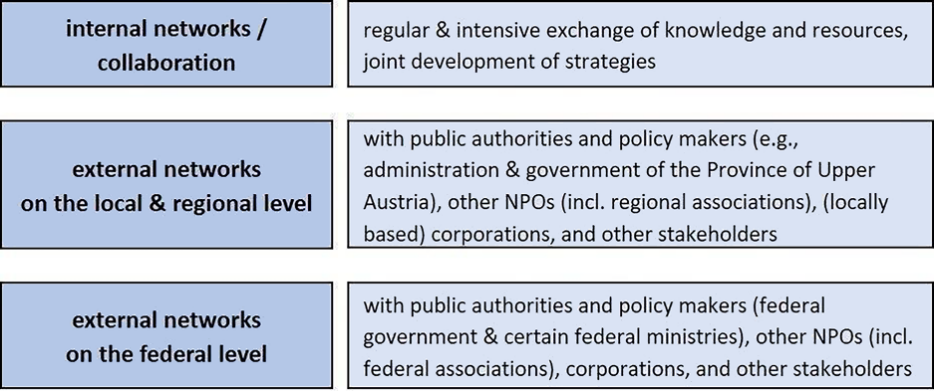
Nonprofits’ supportive relationship networks (own elaboration)

We already mentioned that a (potentially life-threatening) event like a pandemic requires collective responses; besides, *“resilience operates at the interface between an organization and its environment as an ongoing process of protection, assessment, and improvement”* (Mithani 2020, p. 509). With this in mind, we asked our interviewees to what extent they cooperated with other organizations for overcoming the challenges. We present the aggregated results of their answers in this subsection as the social context area is about interactions with others. Overall, the findings point to internal and external forms of collaboration, although we found a broad spectrum from (almost) no interorganizational cooperation (then mainly due to time pressure) to very intensive cooperation, which was primarily cultivated at top management level and largely relied on pre-existing relationships. Fig. 2 gives an overview of the identified supportive relationships (representing social capital) on different levels.

With regard to internal (intra-organizational) collaboration, most NPOs built on an ongoing, intensive, and often cross-regional exchange of knowledge and resources, e.g., between district offices or superordinate regional organizations. Executives also developed common coping strategies (generally oriented strategies or business area-related ones) and, in general, had more regular exchange rounds than usual. As a result, the regional organizational units *“have grown even closer together”* (IP 19).

In terms of external cooperation, we identified connections to various stakeholders. These both formal and informal networks were primarily maintained via telephone and video conferences and facilitated the NPOs’ information and communication options as well as their resource supply. For some NPOs these connections also offered opportunities to influence politics during the pandemic. These relationships take place on the local and regional level, or on the federal level, or partly on both levels. First, we refer to corporate partnerships (with corporate donors or sponsors). Several NPOs got support from businesses, which helped them to obtain scarce goods, to acquire necessary resources or to ensure the operability of their employees. One NPO, for instance, which operates housing facilities for children and teenagers, was able to procure laptops (urgently needed for distance learning) through corporate in-kind donations. The employees of another NPO, who had very long service duties, were provided with food and meals by businesses. Another example were pharmacies facilitating the supply of disinfectants.

Second, a functioning cooperation with various public authorities and policy makers at different federal levels was particularly important for coping with the pandemic, not least because of the close relations and dependencies between social and health care NPOs and public institutions.^17^ On the local and regional level, nonprofit leaders and employees were in close contact with institutions like city authorities, members of the state/provincial governments and health insurance institutions. Many interviewees mentioned a particularly close exchange with politicians and administrative staff of the Province of Upper Austria (e.g., telephone calls with the governor or provincial councilors, or exchange of information with officials of the regional social department) which has also initiated a swap exchange forum for employees (for emergency coping with staff shortages in, e.g., residential facilities). This ongoing exchange of experiences and information was helpful for both sides as the following quote illustrates:

*“(…) sometimes the province and its administration demand things we are not able to do at all (…) a colleague working for the provincial administration recently said: ‘Yes, it’s really great that you call and tell me that, because I’m sitting at my desk and I don’t know what’s going on out there and how it works’.”* (IP 17)

On the federal level, some well networked executives also maintained contacts with responsible decision-makers of the federal chancellery, individual federal ministries, and the federal crisis team (directly or with assistants and press officers). This enabled uncomplicated and early communication and made it possible to draw attention to problems. A few NPOs were involved in the crisis team and able to contribute to the conception of support programs (like the NPO fund).

Third, we identified several ways of cooperation (on different levels) with other NPOs. Although they rarely helped each other with staff or scarce (protective) materials, they exchanged experiences, knowledge, and ideas, they discussed new legal requirements and possible coordinated answers. For example, nonprofit leaders exchanged their knowledge and experiences with executives of other service providers in the same business area (both located in the same state/province and on the federal level), and occasionally some did joint media work. With regard to cross-state cooperation, many interviewees criticized aggravating regulatory differences which one manager called *“all the absurdities of the federal system”* (IP 14). In addition, many interviewees emphasized the relevance of an informal exchange with (well-known or befriended) leaders of other social and health care nonprofits. They did not only share knowledge, ideas and experiences, but also and foremost their worries and everyday problems. That was very helpful *“because others often already have solutions that we are still thinking about, or vice versa because we already have things under control that others are still struggling with. (…) Although we actually are competitors in the same market, we have a very collegial and cooperative relationship. I really appreciate it.”* (IP 12) Last but not least, exchange, knowledge transfer and support within umbrella organizations or associations was also important for many NPOs. This kind of cooperation took place at both the regional and the federal level and was important for the dissemination of up-to-date information (e.g., on new rules and regulations or on possible support grants) and for joint lobbying. The interviewees highlighted the following interest groups on the regional level: “IVS – Interessenvertretung der Sozialunternehmen im psychosozialen- und Behindertenbereich OÖ”, “Sozialplattform Oberösterreich”, “ARGE Mobile Betreuung und Pflege OÖ”, and “ULF – Unabhängiges LandesFreiwilligenzentrum”. Relevant groups at the federal level included: “Sozialwirtschaft Österreich – Verband der österreichischen Sozial- und Gesundheitsunternehmen”, “ÖGKV Bundesarbeitsgemeinschaft (BAG) Mobile Pflege”, and “Fundraising Verband Austria”.

Fourth and last, some of the analyzed NPOs also cooperated with other stakeholders like, e.g., hospitals, school psychologists for supporting children, representatives (of local and national) media, or even district waste associations for joint public relations.

### Synopsis of Contextual Challenges and Resilience Mechanisms

In view of the abundance of pandemic challenges that we identified through our interviews, this final subsection synthesizes contextual challenges as well as the associated abilities for adapting and responding to them. Table 3 provides a comprehensive overview of the identified main challenges and the resilience mechanisms we derived from our analysis for all the four context areas. Those resilient behaviors, resources and capabilities that can be regarded as nonprofit-specific (as they build on their peculiarities in terms of their special value and target system, organizational characteristics and resource mix) have been italicized in the right column of the table.

First, the task context and its core challenges can be summed up as the question how to maintain and extensively adjust business under high uncertainty and dynamic environmental conditions. Since the pandemic represented a new kind of an extreme event in Austria, most NPOs had not established routines or processing patterns for such a comprehensive crisis. Accordingly, it was essential to reflect what is “really” important (in the sense of less is more) and what can be postponed, to be willing to set priorities, to make ad hoc decisions and to install/operate a functioning crisis management. The awareness of simple, almost self-evident things was sharpened and their relevance became clear(er) again (like the importance of calm and clear, timely and target group-oriented communication).

We found that several resources, behaviors and capabilities were instrumental to manage the crisis. First, financial reserves and organizational slack^18^ were essential for avoiding liquidity bottlenecks, for making necessary investments and delivering services despite staff shortages. Without (surplus) resources and, second, the often-exceptional (mission-related, i.e. value-based) commitment and high work ethic from both employed and volunteer workers as well as executives, many NPOs would not have been able to withstand. Third, social capital in terms of supportive networks in general, and in particular some long-standing and trusting relationships were helpful for mitigating the hardships of the pandemic and for adapting to its adversities. Through these internal and external networks NPOs could improve information supply, exchange experiences and knowledge (esp. as public actors often did not provide clear directions), they could also gain and/or secure resources, and last but not least emotionally support each other (in terms of the feeling “common sorry is a sorrow halved”). Fourth, we regard the acceptance of reality and its ambiguities as well as qualities like serenity, optimism, confidence, creativity, flexibility, pragmatism or the ability to improvise^19^ as key for successful absorption, for elasticity and learning. Among other things, these mechanisms enable(d) nonprofit leaders and employees to quickly find practical instead of “perfect” solutions, to make ends meet using existing resources, to be open to innovative approaches and to uphold team spirits under difficult conditions without end in sight.

When dealing with the multifaceted challenges characterizing the temporal context, the following abilities enabled NPOs to overcome these difficulties. First, a high individual resilience of leaders and employees in terms of physical and emotional fitness was often necessary to endure the high pressure, dynamics and ambiguities. In this regard, (at least short) personal time outs for relaxation and individual “energy dispensers” (like hobbies or loved pets) were vital. Second, to share a common value base, work ethic and pronounced commitment to the NPO’s mission seem again to be essential, not least for being able and willing to maintain the necessary energy and effort in the long term given the seemingly never-ending pandemic. Third, the acceptance of reality with its conflicting conditions and an individual capacity to tolerate uncertainty and information overload and to stay calm were instrumental. In addition, flexibility, constant adaptation, improvisation, and pragmatism (incl. courage to gap) were supportive for making quick decisions and coping with pandemic challenges.

Overall, the changed physical environment had various effects on leadership, teambuilding and social issues, health and wellbeing (of both staff and clients), and on work performance. We found that the following mechanisms were helpful for coping with the outlined challenges: financial reserves and organizational slack again enabled absorption of the “pandemic shock”. Sufficient financial resources were essential for maintaining liquidity as well as financing necessary investments and acquisitions (e.g., for protective material or IT equipment). Other organizational excess resources were needed for maintaining operations in particular stressful times where many employees had to cope with an increased workload under partly new and difficult conditions, aggravated by absences of colleagues due to illness, duties of care, or fear. Besides, elasticity and (partially forced) learning were prerequisites for (more or less) quickly switching operations to online formats and designing innovative products or processes (some of which are likely to remain after the pandemic). Especially helpful in this regard were openness to change and new technologies, acting proactively (concerning the implementation of new online services and workflows as well as trying out new IT solutions at an early stage), creativity, courage and pragmatism (for quickly finding perhaps unorthodox, but feasible solutions), and again slack (as, e.g., learning and practicing new IT skills requires resources). Finally, yet importantly, many employees, volunteers and leaders showed an extraordinarily high level of intrinsic motivation, idealism, and sense of duty that also might have helped them to endure the outlined strains since the pandemic outbreak.

Summarized, social context-related challenges were also manifold and shaped by the necessary leadership and cooperation at a distance as well as by the demanding handling of emotions and mental issues. Here, the following mechanisms seem useful. Once more, slack and social capital (in terms of both internal and external supportive relationships) are important as these facilitate quick and creative solutions to new problems as well as (resource, knowledge and emotional) support. In addition, shared visions and values as well as the high levels of intrinsic motivation and the (often) strong work ethic (ethic of care) were helpful for maintaining commitment, internal cohesion, and (emotional) well-being. This implies both reflecting on one’s own mission and a dynamic process of redefining or reinterpreting one’s organizational identity. Generally, several individual/team characteristics seem helpful for getting through the crisis well. These are (partly again): capacity to tolerate uncertainty and accept reality, (individual and shared) optimism, hope, faith, humor, physical and emotional fitness, self-efficacy, confidence (e.g., be a self-confident leader), creativity, and flexibility.

With regard to cooperation, we found several forms of supportive relationship networks on three levels that were all rated as valuable support factors for overcoming the pandemic challenges. Consequently, these (internal and external) relationship networks can be regarded as a relevant resilience mechanism. It should be noted here that the interviewed leaders mostly relied on already existing relations and only rarely on new partnerships. There was simply no time for most managers to initiate new collaborations; in addition, there were fewer networking opportunities due to canceled events and barely possible face-to-face meetings. Hence, the identified relationships can be characterized as often long-term and trustful partnerships that represent(ed) valuable social capital for nonprofits during times of crisis.

Table 3 also illustrates that the identified resilience mechanisms are linked to different levels. They refer to the individual level, the team and the organizational level. As outlined below, most mechanisms are associated with a single level, but some relate to more than one level. Similar to i.a. Branicki et al. (2019) we regard individual as well as team level resilience mechanisms (in addition to organizational mechanisms) as antecedents for organizational resilience and not vice versa. Individual level resilience mechanisms are personality traits, such as pragmatisms, talent for improvisation, flexibility, creativity, openness, etc. Moreover, individual attitudes (e.g., optimism, serenity or humor) represent crucial individual level mechanisms. We also found evidence for personal states, such as intrinsic motivation, work ethos and emotional as well as physical fitness as individual mechanisms that foster organizational resilience. Prominent mechanisms on team level are the strong commitment to the organizational mission in terms of a common sense of direction of NPO-members as well as supportive relationship networks. The latter, though, do not only refer to team level as individual social capital, but also to organizational level as collective social capital (cf. Brass et al., 2004), as Fig. 2 already illustrated. Crucial resilience mechanisms on the organizational level certainly are financial strength and organizational slack. Moreover, it is worth mentioning, that we also found evidence for improvisation as an organizational ability, even if improvisation is primarily allocated to the individual level. Improvisation on the organizational level refers to the ability of (primarily non-emergency) health and social care NPOs to develop a “spontaneous crisis management” or dynamically changing coordination activities respectively.

## Discussion

Our findings show that pandemic challenges are numerous and multifaceted. In comparison to current research (see Sect. 2), many challenges that we identified are similar to those reported in other papers. Hence, one could conclude that in many countries, NPOs are predominantly facing similar hardships. However, the focus of our analysis was on contextual challenges and consequently it gives a more differentiated insight. Additionally, we identified aspects that complement previous studies like, e.g., challenges related to short-time work, onboarding and gender issues.

Concerning resilience mechanisms, several aspects became apparent: first, that most of these behaviors, resources and capabilities advance NPOs’ abilities for adapting and responding to pandemic challenges in more than one context area and thus appear to be mechanisms that are context independent. Second, the identified mechanisms underline the interconnectedness of resilience levels, especially the connection between individual and organizational resilience, and thus the significance of having committed, highly motivated and resilient executives, employees and volunteers for being able to weather not only the pandemic, but any storm. Nonprofit leaders, in particular, contributed to organizational resilience through their high level of commitment, leadership experience, their social capital (consisting of personal relationships they often cultivated over many years), a distinctive eye for the essentials, courage to leave gaps and make quick decisions as well as several resilient qualities like serenity and optimism.

Third, it comes as no surprise that sufficient resources (financial reserves and organizational slack) are essential for organizational resilience as this aspect became already evident during literature review. In addition to tangible factors, the findings by, e.g., Dayson et al. (2021) also highlighted less tangible enabling mechanisms like, fourth, leadership and guiding values. In line with them and other authors (e.g., Witmer and Mellinger 2016), our results emphasize the extraordinary importance of a strong commitment to a NPO’s mission as a shared value base. This aspect clearly represents a NPO-specific resilience mechanism that other (public or for-profit) providers of social and health care services cannot rely on to the same extent. Shared values and visions usually are the strongest driver for volunteer engagement and also a key (intrinsic) motivation for employees so that they often (want or feel obliged to) go the “extra mile” (which again contributes to organizational slack). Consequently, NPOs can often count on a particularly strong commitment and manifold resource support (of leaders, employees, volunteers, but also donors of money or gifts-in-kind). Other NPO peculiarities are also partially reflected in their special relationship networks (esp. the intensive exchange due to their often-federal structure or in NPO-associations) and also their problem-solving skills. Many NPO leaders were/are not detached, but rather close to the base of employees and volunteers (as lay people) who often improvised and creatively developed innovative individual solutions (also because of their high motivation and work ethic).

Finally, our results clearly show that social capital (supportive networks) is also a highly relevant resilience mechanism. Thus, they confirm previous studies like, e.g., those by Witmer and Mellinger (2016), Hutton et al. (2021) or Waerder et al. (2021) who highlight the relevance of different forms of collaborative relations for recovery and adapting to change. Similar to Waerder et al. (2021) for another extreme context (the 2015 refugee crisis), we found, that pre-existing relationships endured the crisis and were often reinforced. Collaboration facilitated the dissemination of information, exchange of expertise, the acquisition of (financial and personal) resources, and it also entailed emotional or psychological support. In terms of the different kinds of social capital, in our study all three (structural capital referring to the extent or width of connections and how you reach others (e.g., via associations); relational capital (referring to the strength of relations); cognitive capital (subsuming similar visions and values)) seem relevant. In sum, responses to the pandemic generally require(d) a range of actions by multiple actors of all sectors. In this respect, the finding that intra- and intersectoral supportive relationships are of special relevance for NPOs also underlines the linkage of different forms of resilience, i.e., that social capital contributes to both organizational and community resilience.

With a view to further implications for theory, we would like to specifically refer to two works. First, Mithani (2020) distinguishes five modes of resilience (cf. Sect. 2). In the context of our study, we regard only three modes as relevant: absorption, elasticity, and learning. His first mode, avoidance, was just not possible for the NPOs participating in our study (and will neither be for most other NPOs in the world facing a pandemic of global proportions). The last one, rejuvenation, implies complete destruction, and thus this mode was not applicable to our sample (however, it might be interesting to conduct another study examining organizations that have gone bankrupt, for instance, and then rejuvenated). Second, Witmer and Mellinger (2016) highlight six organizational characteristics or qualities. Though their focus is on adaptation to funding changes, some of their resilient qualities seem also appropriate for adapting and responding to COVID-19-related challenges. Our findings support the relevance of four of their characteristics/qualities: commitment to mission, improvisation, community reciprocity (as part of supportive networks), and organizational members’ shared perspective of hope and optimism which also implies *“a focus on opportunities instead of an emphasis on barriers and limitations”* (p. 262). These qualities seem to be common in resilient NPOs, regardless of the type of crisis they are facing. In addition, our findings add to those of Witmer and Mellinger (2016) as we identified some further mechanisms that were important for social and healthcare NPOs in the context of the current pandemic (e.g., financial strength and slack, more types of supportive networks as social capital, acceptance of reality and ambiguities, or creativity, serenity and pragmatism). Moreover, when thinking about the variety of definitions of resilience, we advocate conceptions that understand resilience not only as survival or rising up again, but in a more encompassing way; i.e., also as an opportunity to initiate (continuous) processes of transformation, of organizational development, rejuvenation and learning. We consider such conceptions more suitable and sustainable in today’s turbulent environment.

Overall, our study contributes to both ECR and to research on nonprofit resilience. It underlines that context is important and specifically shows how the unprecedented disruptive context of the COVID-19 pandemic presented itself for Austrian NPOs providing social and health care services, which challenges they faced and how they coped with them. The detailed analysis of the situational features and their linkages to related challenges and coping strategies intends to advance (the fragmented and nascent field of) research on extreme contexts, with the aim of supporting NPOs (and perhaps other organizations) in preparing themselves for future pandemics and crises. In addition, we found some resilience mechanisms to be relevant for several context areas (like, e.g., flexibility, shared visions and values or financial strength and organizational slack) which underscores their importance. Furthermore, a comparison of our results with those of other studies (e.g., Hutton et al. 2021; Waerder et al. 2021, or Witmer and Mellinger 2016) on the one hand shows that it also depends on the type of crisis (or extreme context) which modes of resilience and resilience mechanisms are relevant. On the other hand, different studies show several mechanisms to be relevant also in different (partly extreme) contexts. Therefore, it seems legitimate to conclude that these mechanisms (such as social capital in its various forms) can be regarded as universal resilience mechanisms. Last but not least, our study also makes a contribution to previous resilience research by highlighting which resilience mechanisms are NPO-specific (namely first, their shared visions and values that induce high levels of intrinsic motivation, work ethos and commitment to the mission and organization; second, their associated resource strength (including NPO-specific resources and forms of slack); third, their high improvisation and problem-solving skills (due to the high proportion of lay people and basis proximity), and finally, their distinctive supportive networks).

Our study’s implications for practice are aimed at two groups of practitioners. On the one hand, a deeper understanding of pandemic challenges and of resilience mechanisms valuable in this specific disruptive context can be useful for NPOs and their executives to stay viable and capable of renewal. Incorporating and/or expanding the identified resilience mechanisms can help them to better adapt to the hardships of today’s COVID-19 pandemic and probably also to cope with other pandemics or extreme events in the future. In line with Chikwanda (2020) who states that although *“(…) the pandemic has brought many huge uncertainties, it has also been a time (…) to reimagine who and what your organization is about”* (p. 686) we recommend NPOs to reflect on their strengths and mission, to consciously learn lessons from the COVID-19 pandemic, thus seize the opportunities that lie in any crisis and to foster the mechanisms that enable them “to come back stronger and better”.

On the other hand, our study furthermore provides implications for policymakers. Our findings clearly illustrate that both federal and regional governments (can) play a crucial role during crises by supporting NPOs, but also hindering their work. Financial public support was certainly helpful for securing the organizational existence of Austrian NPOs and businesses (and seemed to be quite pronounced and generous compared to other countries). However, Mithani (2020) notes that instruments like bailouts or economic stimulus advance static, but not dynamic resilience. Moreover, public sector practitioners and especially politicians are recommended to streamline the often dysfunctional, complicated and inefficient multiple responsibilities in Austria’s federal system, which further complicated public communications, the implementation of pandemic countermeasures as well as NPOs’ work and coping with the crisis. Finally, it would be especially useful for the viability and resilience of NPOs in social and health care if public funding would allow them to build up some financial reserves and other slack resources. Several of our interviewees and also Meyer et al. (2021) mentioned the increased pressure to be efficient. These authors point out that *“the lack of organizational slack will not only strain public budgets in times of crises, it will also turn CSOs into less innovative and effective agents in the implementation of public welfare policies”* (p. 22). This development is linked to the now widespread establishment of performance-based contracts that are usually very tightly calculated; consequently, they leave NPOs hardly any room for movement, innovativeness and the formation of reserves or surplus resources. Public financiers are recommended to reflect on this development as it should not be in their interest that NPOs’ capacity to manage adversities as well as their ability to develop solutions for pressing social problems gets further weakened.

## Conclusions

The main focus of our study is on contextual challenges that characterize the ongoing pandemic (from the perspective of nonprofit executives), and on resilience mechanisms that help Austrian NPOs to cope with pandemic adversities in the field of social and health care. Table 3 provides a summary answer to both our research questions (see subsection 4.5) and illustrates, on the one hand, the variety of (partly unprecedented) challenges; on the other hand, it displays which resilience mechanisms enabled NPOs to respond to the manifold challenges and adapt to this disruptive extreme context. From all the different forms of resilient behavior, resources and capabilities that were instrumental to NPOs’ adaptation we would like to highlight a few here. Among others, our findings underscore the high relevance of, first, NPOs’ missions or shared values as a common “lighthouse” for orientation, identification, and creation of meaning. Second, social capital in terms of various (mutual) supportive relationship networks were essential for managing the crisis, as well as, third, reserves or excess resources (organizational slack). Fourth, our findings indicate that NPOs are more resilient to pandemic shocks when they are able to be flexible, responsive, adaptive, and innovative; abilities that once again point to the importance of adequate resource endowments. NPOs can adapt more quickly to adversities when they have leaders that are willing to proactively shape change and renew their organizations, routines and services (instead of only reacting when being forced to). Finally, when experiencing an extreme event like today’s pandemic which – especially in its beginning in 2020 – created a lot of fear and emotion, leaders’ ability to instill optimism and courage in their organizations, to cultivate serenity and humor as well as to maintain mutual trust and motivation seems to be vital. In this regard, learning to accept constant change, instability and ambiguities seems to be a difficult, but beneficial endeavor as it is likely that NPOs, their executives, employees, clients, members, volunteers and various other supporters will have to confront other serious crises in the future.

Overall and throughout this research project, we gained the impression that the work of social and health care NPOs “buffered off” a great deal of damage, especially at the beginning and during the peak phases of the pandemic. If most NPOs hadn’t been so resilient, then arguably the impact of the pandemic – including the suffering and death of many people (especially those belonging to vulnerable groups) – would have been much more severe than it was anyway. Many public actors reacted too slowly and/or chaotic, their guidelines and regulations often were insufficient (i.e., unrealistic or impractical). Therefore, it was all the more important for clients (and society as a whole) that most NPOs took the initiative in a situation-elastic manner, that they were creative, flexible, pragmatic and well networked and able to build on the high commitment of their managers, employees, and supporters. Thus, they quickly found workable solutions and took on responsibility for their beneficiaries and community in an extremely difficult situation that at once demanded everything of themselves. Without them, the care and support of many people in need would no longer have been guaranteed. In sum, NPOs made significant contributions to social life and cohesion, and clearly contributed to community resilience. Consequently, we consider it as imperative to further enhance understanding of NPOs’ organizational resilience and to (also politically) foster their abilities to adapt and respond to challenges as well as to rejuvenate.

As with all empirical approaches, there are some limitations to this study. We acknowledge that its qualitative design based on 33 interviews with leaders of large Austrian NPOs in social and health care does not allow us to make generalizations of our findings. Besides, they may not be transferred one-to-one to, e.g., small NPOs or those active in other fields of activities (like sports or culture). As we captured the lived experiences and subjective perceptions of executives, of course, bias is possible. Our analysis builds on self-reported and partly retrospective views; thus, asking interviewees about their experiences might lead to inaccuracies caused by recall-errors as well as hindsight and attributional bias (Huber and Power 1985). Also, the interview setting and individual question styles (e.g., time pressure of some leaders or different intensities of questioning) can result in response bias. However, some limitations of our inquiry at the same time offer opportunities for future research. For instance, as (individual and organizational) resilience is influenced by national culture (Fietz et al. 2021), our study could be replicated in other countries in order to make comparisons and investigate cultural effects on resilience. Comparative studies could also address differences concerning the impact of the COVID-19 pandemic on NPOs in different parts of the world (esp. between countries in the global North and South). Further research may also consider comparing resilience mechanisms of large and small NPOs and between different fields of activities for analyzing the influence of such (and other) organizational characteristics. Finally, both a deeper and broader exploration of each of the identified resilience mechanisms as well as the interactions between the different levels of resilience (individual, team, organizational, and community) seems worthwhile to advance understanding of nonprofit resilience. This could enable both NPOs and their communities to better prepare for future adversities.

## Footnotes

1 Crisis management focuses on the ability of systems to function normally again after a disruption (Williams et al. 2017); ECR deals with risky, emergency and disrupted contexts (cf. Sect. 2.3) that pose a direct risk to life and limb (Hällgren et al. 2018). Thus, ECR’s focus is different as it deals with certain types of crises (but not with less immediately existential ones such as, e.g., reputation crises).

2 Viewing resilience as a process (instead of as an outcome of organizational recovery from hardship) focusses on how organizations actually “do” resilience. This also involves their managers’ resilience as individual resources and leadership function (Witmer and Mellinger 2016) as organizational and individual resilience are interconnected (Hillmann and Guenther 2020).

3 Bell et al. (2018) attribute (one or more) contextual features to extreme teams that are atypical in level or kind (e.g., involving extreme time pressure or danger). In addition, poor performance has serious consequences for such teams (impairing the health or well-being of team members or clients). Our literature review on pandemic challenges presented above already made it clear that the work of social and health care NPOs and their employees (e.g., in nursing homes) shows parallels to these features of extreme teams. From our point of view, the work environments of such NPOs during the pandemic can be classified as partly extreme and partly conventional, thus as hybrid, as they show both the characteristics of a conventional and of a high-consequence work environment. Moreover, Bell et al. (2018) also consider their (broad) approach for organizing context *“as applicable to more conventional organizational teams”* (p. 2742).

4 This kind of (actual and potential) resources was famously coined by Bourdieu (1986) as one of four forms of capital: economic capital (money or material things), cultural capital (like educational qualifications or culturally valued tastes and preferences), symbolic capital (as authority that comes with status and prestige, which for Bourdieu is the most powerful form), and social capital made up of memberships in a group, networks or “connections”. These (power) resources are unequally distributed, a prerequisite of habitus formation and shape the possibilities for action or practice. Besides, they are convertible and exchanged between individuals, groups, or social classes (Bourdieu 1977, 1986; Ebrahim 2005).

5 The authors further differentiate between proactive and reactive resilience and found that structural capital (which refers to linkages, i.e. who you reach and how you reach them) can predict proactive resilience while relational capital (which entails the strength of a relationship) improves reactive organizational resilience. They found no significant influence of cognitive capital (representing similar visions, goals, and values within a social system).

6 Of course, many services in social and health care are also offered by other (namely public and/or private for-profit) providers. The reason why we focus on NPOs and not on social and health care in general, is that such a broad(er) institutional focus would significantly increase the heterogeneity of organizations under investigation. From our point of view, such a diversity would make meaningful comparisons more difficult, since both the characteristics and (partly) the context of NPOs as well as of public institutions and private businesses in the social and health sector are different.

7 Since top executives are often difficult to reach and time-constrained (Ma et al. 2021), the authors partly used their personal networks (both private and professional contacts) to gain access and to motivate executives to participate. Fortunately, many leaders were highly interested in the topic (a later “weariness” with the pandemic was not noticeable at the time) and several participants saw the interview as a good opportunity for reflection.

8 Open questions are often well suited for elite actors as they are mostly able to respond at length and proactively provide explanations and examples. However, it should be considered that many experienced executives also tend towards dominant behavior and impression management (cf. Ma et al. 2021).

9 Concerning resilience mechanisms, we combined inductive and deductive coding as here we could build on previous (resilience) literature by Mithani (2020), Hillmann and Guenther (2020) and partly also by Witmer and Mellinger (2016) in terms of selected NPO-specific (resilient) qualities.

10 Table 3 in Sect. 4.5 shows the final set of (mainly inductively generated) subcategories of the main challenges we identified and of the resilience mechanisms (already grouped according to the four context areas).

11 We conducted the interviews in German. All quotes referred to in the text are translations by the authors.

12 All interviewees were anonymized and consequently we assigned a number to each interview partner (IP).

13 The “NPO fund” or “NPO-Unterstützungsfonds” represents a fixed-cost subsidy for NPOs established in 2020 by the Austrian federal government (cf. BMKÖS 2021; Meyer et al. 2021).

14 For information on the Austrian short-time work program (“Covid-19-Kurzarbeit”) cf. AMS Österreich (2021).

15 These contradictory measures are based on the fact that in some service areas (like in residential facilities) manpower requirements increased whereas in others (e.g., workshops, charity shops or day care centers) shutdowns were necessary or even mandated.

16 Of course, home office was not possible for employees in specific service areas, like, e.g., caring for clients in residential facilities. Many top managers also said that they returned to work in the office after a relatively short time and despite the ongoing pandemic, because there they often had better access to work equipment and resources. Besides, some missed the feeling of togetherness through personal exchange which also facilitates communication and coordination. Moreover, they often consciously wanted to be present for their employees.

17 Some NPOs (like, e.g., the Austrian Red Cross) have been well connected with public institutions (both on the regional and the federal level) for a long time and have played a vital role in managing the current pandemic and also other crises together with public sector entities.

18 Organizational slack refers to any organizational surplus of resources. Such buffers, reserves or excess resources open up room for maneuver for a company or NPO (cf. Krcal 2012 who gives an informative overview on the topic). We base our understanding of the term on the conception by Bourgeois III (1981). He defines slack as *“the resource that enables an organization both to adjust to gross shifts in the external environment with minimal trauma, and to experiment with new postures in relation to that environment, either through new product introductions or through innovations in management style (…)”* (p. 31).

19 We understand organizational improvisation in line with Witmer and Mellinger (2016) as *“the ability to utilize existing resources, such as adapting physical space and sharing staff between programs”* (p. 260).
